# Clonal ST131-*H*22 *Escherichia coli* strains from a healthy pig and a human urinary tract infection carry highly similar resistance and virulence plasmids

**DOI:** 10.1099/mgen.0.000295

**Published:** 2019-09-17

**Authors:** Cameron J. Reid, Jessica McKinnon, Steven P. Djordjevic

**Affiliations:** ^1^​ The i3 institute, University of Technology Sydney, Ultimo, NSW 2007, Australia

**Keywords:** HI2, ColV, porcine commensal *E. coli*, ExPEC, ST131, fimH22, H22

## Abstract

The interplay between food production animals, humans and the environment with respect to the transmission of drug-resistant pathogens is widely debated and poorly understood. Pandemic uropathogenic *Escherichia coli* ST131-H30*Rx*, with conserved fluoroquinolone and cephalosporin resistance, are not frequently identified in animals. However, the phylogenetic precursor lineage ST131-H22 in animals and associated meat products is being reported with increasing frequency. Here we characterized two highly related ST131-H22 strains, one from a healthy pig and the other from a human infection (in 2007 and 2009, respectively). We used both long and short genome sequencing and compared them to ST131-H22 genome sequences available in public repositories. Even within the context of H22 strains, the two strains in question were highly related, separated by only 20 core SNPs. Furthermore, they were closely related to a faecal strain isolated in 2010 from a geographically distinct, healthy human in New South Wales, Australia. The porcine and hospital strains carried highly similar HI2-ST3 multidrug resistant plasmids with differences in the hospital strain arising due to IS-mediated insertions and rearrangements. Near identical ColV plasmids were also present in both strains, further supporting their shared evolutionary history. This work highlights the importance of adopting a One Health approach to genomic surveillance to gain insights into pathogen evolution and spread.

## Data Summary

1. Short and long reads for both F2_14D and 2009_36 have been uploaded to SRA under BioProject accession number PRJNA508590.

2. Annotated sequences of pF2_14D_F, p2009_36_F, p2009_36_HI2 and pF2_14D_HI2 are available with GenBank accession numbers MK461928, MK461929, MK461930 and MK461931, respectively.

**Impact Statement**

This article provides insight into the global population structure and accessory elements of *Escherichia coli* ST131-*H*22. The genetic relatedness of the two strains characterized here by whole genome sequencing, despite their temporal and geographical disparity, raises further questions about the potential occurrence of food-borne urinary tract infections. This study provides a starting point for examining this hypothesis in Australia, a country where sound antimicrobial stewardship has been shown to limit carriage of antibiotics of human clinical significance in food animal production. The HI2-ST3 multidrug resistance plasmids carried by the two strains belong to a group of regionally disseminated drug resistance plasmids, but were differentiated from other sequences by their carriage of a mobile element encoding heavy metal resistance genes. This has implications for the use of in-feed metals in swine production, as drug resistance may be co-selected in the absence of antimicrobial selection pressure. Similarly, the co-carriage of ColV virulence plasmids raises the possibility of co-selection of hyper-virulent strains of *E. coli* as a by-product of food animal production.

## Introduction

Multi-drug-resistant (MDR) extra-intestinal pathogenic *Escherichia coli* (ExPEC) that cause urinary tract infections (UTIs), pyelonephritis and urosepsis represent a significant healthcare burden worldwide [1]. Whilst a diversity of *E. coli* clones representing various multi-locus sequence types may cause extra-intestinal infections [2], much recent study has focused on the globally disseminated ST131 *H*30*Rx* sublineage of ST131. ST131 *H*30*Rx* causes a significant proportion of hospital and community-acquired urine and blood-related infections and is resistant to several last-line clinical antibiotics such as fluoroquinolones and extended-spectrum beta-lactams [3] conferred by expression of CTX-M-type extended-spectrum beta-lactamase (ESBL). The literature is heavily biased in its description of *Enterobacteriaceae* that display resistance to these and other last-line drugs of clinical importance to human health. ST131 as a clonal group exhibits subpopulation clonal structure that correlates with carriage of different alleles of the fimbrial adhesin gene *fimH*. FimH facilitates adherence to uroepithelium, bladder cell invasion and establishment of intracellular bacterial communities [4]. Three major *fimH* alleles, *fimH*30*, H*22 and *H*41, dominate ST131 phylogeny with the latter two being basal to *H*30 [5, 6].

*H*22 strains, which are purported to have given rise to *H*30*Rx*, are underrepresented in the literature and sequence databases. However, *H*22 strains also cause serious extra-intestinal infections [7] and ignorance of these ancestral, yet extant lineages obscures the factors that established ST131 before the *H*30 lineage acquired SNPs conferring fluoroquinolone resistance and ESBL genes. Increasing evidence indicates that ST131, and *H*22 in particular, are successful commensals [8]. A recent study showed that *H*22 strains isolated from healthy humans might have an advantage in the human gut due to use of gluconate as a carbon source and an enhanced ability to form biofilms [9]. Furthermore, the global distribution of *H*22 and carriage of plasmids that influence intestinal fitness supports the contention that widespread faecal carriage was the initial reason for their success [10].

In addition to human carriage, *H*22 has been reported in wild seals and poultry meat, and was recently identified carrying colistin resistance determinant *mcr1* in swine from Spain [10–12]. A growing body of literature suggests that intensive animal production in particular plays a role in the selection and emergence of drug-resistant ExPEC, although more work needs to be done in order to understand the extent to which this occurs [10, 13–17]. One recent study explicitly implicated *H*22 strains as food-borne pathogens contaminating poultry meat and arising in human UTIs in Flagstaff, Arizona. This was the largest study of *H*22 genome sequences to date and found that some UTI strains clustered with poultry strains based on whole genome phylogeny. The authors noted repetitive acquisition of ColV plasmids in different branches of the phylogeny [10]. ColV plasmids are implicated in the carriage of virulence genes associated with avian pathogenic *E. coli* (APEC) and the ability to cause disease in different animal models [18, 19]. The presence of ST131 sub-clones in animals may be attributable to humans in the first instance, although this does not exclude animals from playing a role in its ongoing evolution and dissemination, as seen in a Spanish study in which colistin resistance has clearly emerged in response to the use of this antimicrobial in swine production [12]. Regardless of origin, the presence of a human pathogen in animals is a concern as resistance, virulence and fitness traits encoded on mobile DNA that circulate in animal production may drive the evolution of even more severe human pathogens.

In Australia, there is a limited but important body of data describing the genomic epidemiology of *E. coli* from swine and poultry [18, 20]. Our studies and those of others show that stewardship practices have been successful in limiting carriage of resistance genes encoding resistance to clinically important antibiotics, but more comprehensive studies are needed [21, 22]. Far larger collections of systematically sampled, temporally and geographically related populations of strains from humans, animals and the environment need to be considered using a whole genome sequencing approach. SNP-based core genome phylogenies and long read sequencing characterization of resistance and virulence plasmids and other mobile elements provide superior resolution to multi-locus sequence typing (MLST) and screening of individual genes alone. In the absence of these large datasets, insights can still be gathered with the aforementioned methods and public databases. By understanding H22 and other established pre-pandemic lineages we may be able to predict future pathogen expansion events.

Here we characterized and compared two *H*22 strains from disparate sources in New South Wales (NSW), Australia. F2_14D was isolated in 2007 from a healthy piglet at a rural production facility and 2009_36 was isolated in 2009 from a human UTI at a suburban hospital. More than 250 km separated the hospital from the farm. We aimed to compare their antimicrobial resistance genes (ARGs), virulence-associated genes (VAGs) and plasmid types to a global collection of *H*22 strains and perform long-read sequencing to characterize the plasmids they carried.

## Methods

### Strains and sequences used in this study

*E. coli* strain F2_14D was isolated from a weaned piglet by faecal swab in 2007 from an intensive production system with a history of extensive neomycin use for treatment of enterotoxigenic *E. coli* (ETEC) outbreaks. More information on the collection has previously been reported [20]. 2009_36 was isolated from catheter stream urine of a patient with a UTI at the Sydney Adventist Hospital in Sydney in 2009. Enterobase (http://enterobase.warwick.ac.uk/species/index/ecoli) was queried for ST131 *fimH*22 strains (accessed 16 May 2019) and sequences with metadata for source niche and type, year of isolation, country and continent were downloaded from SRA using parallel-fastq-dump v0.6.3 (https://github.com/rvalieris/parallel-fastq-dump). Some manual editing of source information was required to create consistency, including addition of information for 41 publicly available sequences (25 poultry meat, 16 human) previously described by Liu *et al.,* and this information was drawn from the supplementary data of their publication. Both raw and processed Enterobase data are available in Tables S1 and S4 (available in the online version of this article), respectively. The final collection of publicly available sequences (*n*=280) was split into Source Niches, each encompassing Source Types: Food (beef, poultry meat, pork; *n*=70), Human (clinical, faecal, misc; *n*=158), Livestock (bovine, chicken, turkey, swine; *n*=37), Environment (soil, water; *n*=6), Wild Animal (marine mammal, gull; *n*=3) and Companion Animal (canine, feline; *n*=6). Both the raw Illumina reads and complete genome of JJ1897, an ST131 *H*22 isolated from a human infection, were downloaded for gene screening and use as a reference genome respectively. The 282 sequences used in this study and their accession numbers are available in Table S1.

### DNA isolation, sequencing and assembly

DNA from both strains was isolated, quantified and sequenced on an Illumina HiSeq 2500 v4 sequencer as previously described [20]. Illumina short reads have been uploaded to SRA. Accession numbers are available in Table S1. DNA was also isolated from both strains for long-read sequencing by phenol/chloroform extraction (full method available in File S1) and quantified by a Qubit dsDNA HS assay (Thermo Fisher Scientific) as previously described [20]. The suitability of the DNA for long-read sequencing was assessed by electrophoresis using a 0.8 % agarose gel, run at 10 V for 16 h. DNA purity was confirmed by using a Nanodrop device (Thermo Fisher Scientific). Libraries were prepared for long-read sequencing using the Oxford Nanopore Technologies (ONT) 1D ligation sequencing kit (SQK-LSK108) with the native barcoding expansion kit (EXP-NBD103). Several modifications were made to the ONT protocol to maximize read length and throughput, including those described by Wick *et al.* [23]. In addition, we used 7.5 µg of starting DNA from each isolate and performed the DNA purification steps using SPRIselect beads (Beckman Coulter). Resuspension of SPRIselect beads was carried out at higher than usual temperatures (50 °C after end repair and 37 °C after adapter ligation) to promote efficient elution of the DNA into solution. The final library containing 4.4 µg DNA was loaded onto an ONT MinION instrument with a FLO-MIN106 (R9.4) flow cell and run for 48 h as per the manufacturer’s instructions. Raw fast5 files were base-called with Albacore v2.3.3 (ONT) and de-multiplexed with Porechop v0.2.3 (https://github.com/rrwick/Porechop) with default settings. Reads were then subsampled to 500 Mbp with Filtlong v0.2.0 with default settings and a minimum read length of 2000 bp. Unicycler v0.4.6 [23] hybrid assembly was performed with default settings using both Illumina raw reads and ONT subsampled reads. Contigs less than 1000 bp were excluded from the final assembly. Assembly graphs viewed in Bandage [24] revealed the chromosome of 2009_36 was fragmented into 118 contigs with a continuous yet unresolvable assembly path, although two circular contigs of 278 665 and 143 671 bp were assembled (final contigs: 120). F2_14D consisted of a single contig linear chromosome and a circular contig of 274 883 bp. Three further contigs with a continuous graph path were joined and exported from Bandage in fasta format, forming the third circular replicon 139 372 bp in length (final contigs: 3). See Table S1 for all SRA and GenBank accession numbers associated with this study.

### Phylogenetic analysis

In order to generate a global ST131-*H*22 core SNP phylogeny, Illumina raw reads were aligned to the complete genome of *H*22 strain JJ1897 (gb|CP013837.1) using Snippy v4.1.0 (https://github.com/tseemann/snippy). snippy-core was then used to generate an alignment of all strains. The snippy-core full alignment was recombination-filtered with Gubbins [25] and SNPs were identified with snp-sites v2.4.0, resulting in an alignment of 3720 core variable sites [26]. FastTree2 v2.1.10 was then used with default settings to produce a maximum-likelihood phylogenetic tree from the alignment using a generalized time-reversible (GTR) nucleotide substitution model [27]. The tree was visualized with strain metadata in iTOL v4.2.3 [28]. The tree was also visualized alongside a gene-screening heatmap in R v3.3.1 with ggtree v3.6 [29]. Pairwise SNPs between all strains were extracted from the core alignment using snp-dists v0.6 (https://github.com/tseemann/snp-dists). The tree, alignment from Gubbins, final core alignment generated using snp-sites and the pairwise SNP table are available at https://github.com/CJREID/ST131-H22_supporting_data.

### Gene screening

All *H*22 sequences were screened for ARGs, VAGs, plasmid replicons, O and H antigen genes, and pMLST alleles using ARIBA v2.10.1 [30] and ResFinder, PlasmidFinder, VirulenceFinder and SerotypeFinder databases available from the Center for Genomic Epidemiology (http://www.genomicepidemiology.org/) [31–34]. A custom database of additional genes not present in the aforementioned databases was also used. This database is available at https://github.com/CJREID/ST131-H22_supporting_data. Criteria for ‘ColV-positive’ strains was defined as carriage of at least one gene from four or more of the following six groups: (i) *cvi, cvaA, cvaB, cvaC*; (ii) *iroN*; (iii) *iucD, iutA*; (iv) *etsA*; (v) *ompT*; and (vi) *sitA.*

### Plasmid annotation and visualization

Circular plasmid contigs resulting from the Unicycler assembly were annotated with RASTtk via the Pathosystems Resource Integration Centre’s (PATRIC) online Bacterial Bioinformatics Resource Centre (https://patricbrc.org/). This automated annotation was then imported in GenBank format into SnapGene 4.1.9 (GSL Biotech) and manual curation was performed with blastn (https://blast.ncbi.nlm.nih.gov/) and the previously mentioned gene databases. NCBI blastn was also used to select complete plasmids for comparison with our sequence. For F plasmids, mobile elements and transposons were removed from the backbone of p2009_36_F and the backbone sequence was used as a query to GenBank. Complete plasmid sequences with 100 % coverage and ≥97 % identity were downloaded for comparison (*n*=13). For HI2 plasmids, the *smr0018* and *smr0199* alleles were used as a query to identify and download all publicly available HI2-ST3 plasmids (*n*=41) (accessed 25 October 2018). Details of downloaded plasmids are available in Tables S2 and S3. blast Ring Image Generator (BRIG) v0.95 was used with default settings to compare our plasmid sequences to publicly available sequences [35]. These figures were then combined with the SnapGene-generated plasmid maps to visualize similarities and differences.

## Results and Discussion

### F2_14D and 2009_36 in the context of ST131-H22 global phylogeny

Routine preliminary phylogenetic and gene screening analysis (data not shown) of short read sequence data undertaken with isolates from the Sydney Adventist Hospital and commensal *E. coli* from healthy swine in NSW [20] indicated that two ST131 strains from these collections were closely related and had similar accessory genes despite no epidemiological data to link them. These strains were 2009_36, isolated from the catheter of a human with UTI in 2009, and F2_14D, isolated from a faecal swab of a healthy piglet in 2007.

**Fig. 1.**
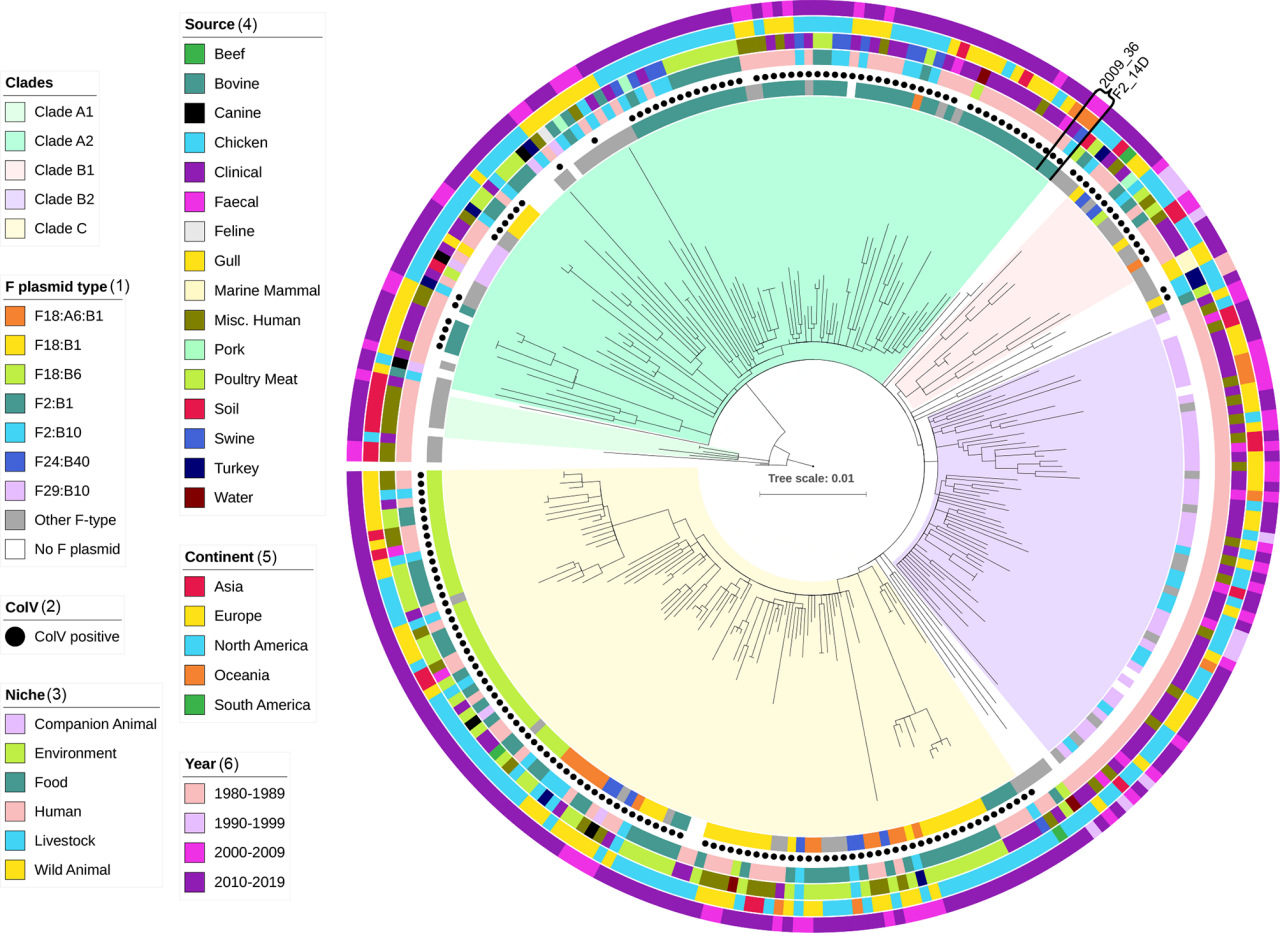
Maximum-likelihood tree of 282 ST131-*H*22 sequences derived from a core genome alignment of 3720 variable sites against reference genome JJ1897. Coloured bars and black dots show metadata associated with each strain.

In order to determine how similar these two strains were at a core genome level we performed SNP-based alignment with snippy, using the closed genome of ST131-H22 JJ1897 as a reference and 280 publicly available *H*22 strains. The core genome alignment used to build the tree consisted of 3720 conserved variable sites (Fig. 1).

The total collection of 282 sequences was from food (*n*=70), human (*n*=159), livestock (*n*=38), environment (*n*=6), wild animal (*n*=3) and companion animal (*n*=6) sources, spanning 1982–2019, five continents and 17 countries (Tables 1 and S1). The collection was unsurprisingly dominated by human clinical sequences from Europe and North America, highlighting the human bias in infectious disease research and the persistence of ST131-*H*22 infections despite the literary dominance of *H*30. Food source sequences were mostly from poultry meat, a well-established source of potentially infectious and drug-resistant *E. coli* [10]. It is important that there is an increase in sequenced strains from livestock as well as from healthy human faecal samples so that holistic genomic epidemiological studies can properly examine the relationships between *E. coli* (from livestock, food and the human gut) and human infections. The low number of human faecal-derived strains in particular represents a significant knowledge gap, as human faecal carriage of *H*22 is previously reported and faecal matter is typically the immediate source of extra-intestinal infections caused by *E. coli* [9, 36, 37].

**Table 1.** Summary of 282 ST131-*H*22 *E. coli* sequences used in this study

	**Asia**	**Europe**	**North America**	**Oceania**	**South America**	**Total**
**Companion animal**		**2**	**4**			**6**
Canine		1	4			**5**
Feline		1				**1**
**Environment**		**2**	**4**			**6**
Soil			2			**2**
Water		2	2			**4**
**Food**		**15**	**55**			**70**
Beef			1			**1**
Pork		2	1			**3**
Poultry meat		13	53			**66**
**Human**	**25**	**67**	**57**	**8**	**2**	**159**
Clinical	4	40	43	5		**92**
Faecal	6		3	2		**11**
Other	15	27	11	1	2	**56**
**Livestock**		**13**	**23**	**2**		**38**
Bovine		4				**4**
Chicken		7	5	1		**13**
Swine		1	11	1		**13**
Turkey		1	7			**8**
**Wild animal**		**2**	**1**			**3**
Gull			1			**1**
Marine mammal		2				**2**
**Total**	**25**	**101**	**144**	**10**	**2**	**282**

The tree exhibited three major clades, designated A2, B2 and C, as well as two minor clades A1 and B1 (Fig. 1). A number of sequences were present between these clades. Clades A2, B1 and C were characterized by a mixture of human, livestock and food origin sequences from Europe and North America. Clade B2 notably contained only human origin sequences, faecal, clinical and undetermined, from all continents except South America. It is likely that this clade represents a lineage that has specifically adapted to humans as opposed to a sampling peculiarity, given its intercontinental distribution and the fact that source overlap was present in the other clades.

*In silico* serotype screening determined 253 of 282 sequences to be O25:H4 or O25*:H4, the asterisk noting SNP variations from the O25 reference sequence. In total, 21 strains were O- or H-non-typeable: ONT:H4 (*n*=16), ONT:HNT (*n*=1), O25*:HNT (*n*=3) and O25:HNT (*n*=1). Five strains had ambiguous O-types or H-types whereby the result indicates more than one O or H allele (Table S1). The predominance of O25:H4 or similar is concordant with previously reported ST131-*H*22 strains [3]. Three strains unexpectedly exhibited different types O15:H4 (*n*=2) and O2:H4 (*n*=1). Whilst *E. coli* are capable of switching serotypes, this change within a clonal group such as ST131 is worthy of further investigation, although it is evidently uncommon.

Strains 2009_36 and F2_14D were present in clade A2 and separated by 20 SNPs. Interestingly, an Australian human faecal strain 11.3-R3 from 2010 was also closely related, separated by 24 SNPs from both 2009_36 and F2_14D. Whilst more examples of this lineage from animals and humans are required to understand potential reservoirs, it is remarkable that three strains from randomly sampled collections are so closely related. More sequences from diverse sources in Australia are required to determine whether this lineage is common in the region. The fact that F2_14D was the only ST131 in a collection of 68 isolates from 21 pigs suggests its presence in swine is infrequent, although more extensive studies of porcine commensal *E. coli* are required to explore this hypothesis [20].

### ST131-H22 virulence profiles appear to reflect their phylogeny

In order to determine the repertoire of accessory elements in *H*22 strains and examine any patterns in the context of the phylogeny, we screened for virulence, resistance, plasmid-associated and common mobile element genes and mapped them against the core genome phylogeny (Fig. S1). Strains carried 25 VAGs on average with a range of 10 to 43. The uropathogenic-specific protein gene *usp* was the only VAG present in all 282 strains. Iron acquisition genes *fyuA* (*n*=277, 98 %), *sitA* (*n*=274, 97 %)*, irp2* (*n*=270, 96 %)*, iucD* (*n*=226, 80 %) and *iutA* (*n*=220, 78 %) were abundant, as were immune evasion/protectin genes *kpsMTII* (*n*=259, 92 %)*, traT* (*n*=262, 93 %) and *iss* (*n*=277, 98 %) and invasion of brain endothelium gene *ibeA* (*n*=275, 98 %) [38–45]. Each clade had noticeably different VAG profiles, although there was still variation within clades (Fig. S1).

### ColV carriage is associated with phylogeny and multiple F plasmids

Clades A2, B1 and C were notable for their carriage of ColV plasmid-associated genes*,* which were absent from clade B2. A total of 176 strains were considered ColV-positive in the collection (62 %). ColV plasmids are typically F-type plasmids, strongly associated with APEC where they provide an advantage in the gut and virulence in extra-intestinal sites they infect [18, 46]. Furthermore, they have been identified in human commensals carrying ARGs and human urine and blood infections, indicating they may play a similar role here [10, 19, 36, 46, 47]. F-plasmid pMLST type and ColV presence/absence appears to be related to evolutionary clades within ST131-*H*22 (Fig. 1). A total of 23 F-plasmid pMLST combinations corresponded to carriage of ColV genes in the collection with the most common being F2:B1 (*n*=57/176, 32 %), F18:B6 (*n*=37, 21 %), F18:B1 (*n*=28, 16 %) and F18:A6:B1 (*n*=16, 9 %). Clade A2 was dominated by F2:B1, whilst F18 variants were characteristic of clade C. Clade B1 contained F18 variants and F24:B40. F plasmids were also present in most ColV-negative strains (*n*=89/106, 84%), although they were of distinct pMLST types, predominantly F29:B10 (*n*=39/106, 37 %) and F2:B10 (*n*=9, 8 %). ColV-negative strains carrying F29:B10 plasmids were characteristic of the human-only clade B2 and have previously been identified in *H*22 carrying ColIa genes as opposed to ColV [48]. By contrast, ColV-positive clades traversed human, food and livestock niches, with poultry meat being a notable source (*n*=17/61, 35 %).

The presence of 23 pMLST types among ColV-positive strains supports Liu’s contention that ColV plasmids have been acquired multiple times by different branches of the *H*22 phylogeny and suggests that their estimation of at least six acquisition events is likely to be conservative [10]. However, the presence of 23 F-types does not necessarily indicate 23 separate acquisition events as a number of allelic combinations were derivatives of others such as F18:A6:B1 and F18:B1 in clade C. Further analysis is required to determine if this represents two separate acquisition events or one acquisition followed by loss of the A6 replicon. The carriage of ColV plasmids in these lineages suggests they have been exposed and adapted to niches where these plasmids are common such as poultry production systems and the human gut [10, 18, 46].

The carriage of ColV plasmids by clades with diverse sources and absence of ColV plasmids in the human-specific clade was intriguing. First, it suggests that ColV plasmids may confer broad fitness characteristics in a variety of niches, although they are not necessary to cause human disease. Second, given the known association of ColV plasmids and poultry, ColV carriage may indicate either a poultry origin or some historical contact between a given lineage and poultry production. Further genomic epidemiological studies tracking ColV plasmids to understand their reservoirs and routes of transmission are required to explore this hypothesis.

### Antimicrobial resistance genes in ST131-H22

Fourteen strains carried no ARGs whilst the remainder carried between one and 16 at an average of four per strain. ARGs conferring resistance to aminoglycosides (*aadA1,* *n*=39, 14 %), penicillin (*bla*_TEM-1B_, *n*=67, 28 %; *bla*_TEM-1C_, *n*=50, 18 %), streptomycin (*strA,* *n*=44, 16 %; *strB,* *n*=46, 16 %), sulphonamides (*sul1,* *n*=53, 19 %; *sul2*, *n*=45, 16 %) and tetracycline (*tetA,* *n*=79, 28 %; *tetB,* *n*=41, 15 %) were most common. These genes are frequently encountered in commensal and pathogenic *E. coli* that inhabit the gut of swine [20, 49, 50] and poultry in Australia [18]. Furthermore, IS*26*-flanked transposons carrying these genes, such as Tn*6029* and Tn*6026,* are frequently encountered in food animals and humans in Australia on diverse plasmid backbones [36, 51–55] and in the chromosome [56]. It is notable that these transposons are also present on virulence plasmids including ColV, IncI and IncZ plasmids in human intestinal pathogenic *E. coli* in Australia [36, 54, 57]. The class 1 integrase gene *intI1* was present in 61 strains (22 %), all of which carried multiple ARGs, consistent with the association between class 1 integrons and multidrug resistance [58]. Strains carrying ARGs typically carried a non-F type plasmid replicon. This indicates that the history of antimicrobial exposure for each strain remains important within *H*22 and they continue to acquire diverse plasmid types. This is highlighted by the presence of ESBL and colistin resistance genes also present in a subset of strains, albeit a low number. ESBL genes identified included *bla*_CTX-M-9_ (*n*=10, 4 %), *bla*_CTX-M-1_ (*n*=8, 3%) and *bla*_CMY-2_ (*n*=35, 12 %) (Fig. S1 and Table S1). Mobile colistin resistance determinants *mcr-1* (*n*=4, 1%) and *mcr-9* (*n*=8, 3 %) were also present. Further selection of ESBL and colistin genes in *H22* strains could lead to an increased prevalence in hospital infections where *H*30 strains currently dominate.

### Plasmids in 2009_36 and F2_14D

We used long-read sequencing to determine the similarity between the plasmids carried by 2009_36 and F2_14D. This confirmed each strain carried an F2:B1 ColV plasmid and an HI2-ST3 plasmid. These plasmids were designated p2009_36_F, pF2_14D_F, p2009_36_HI2 and pF2_14D_HI2.

**Fig. 2.**
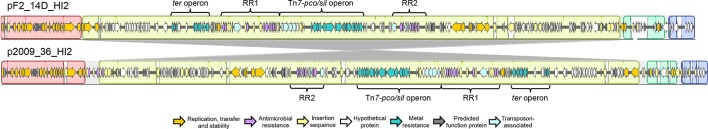
Mauve comparison of pF2_14D_HI2 and p2009_36_HI2. Grey colouring between plasmids indicates sequence inversion. Coloured arrows represent genes and their functions.

**Fig. 3.**
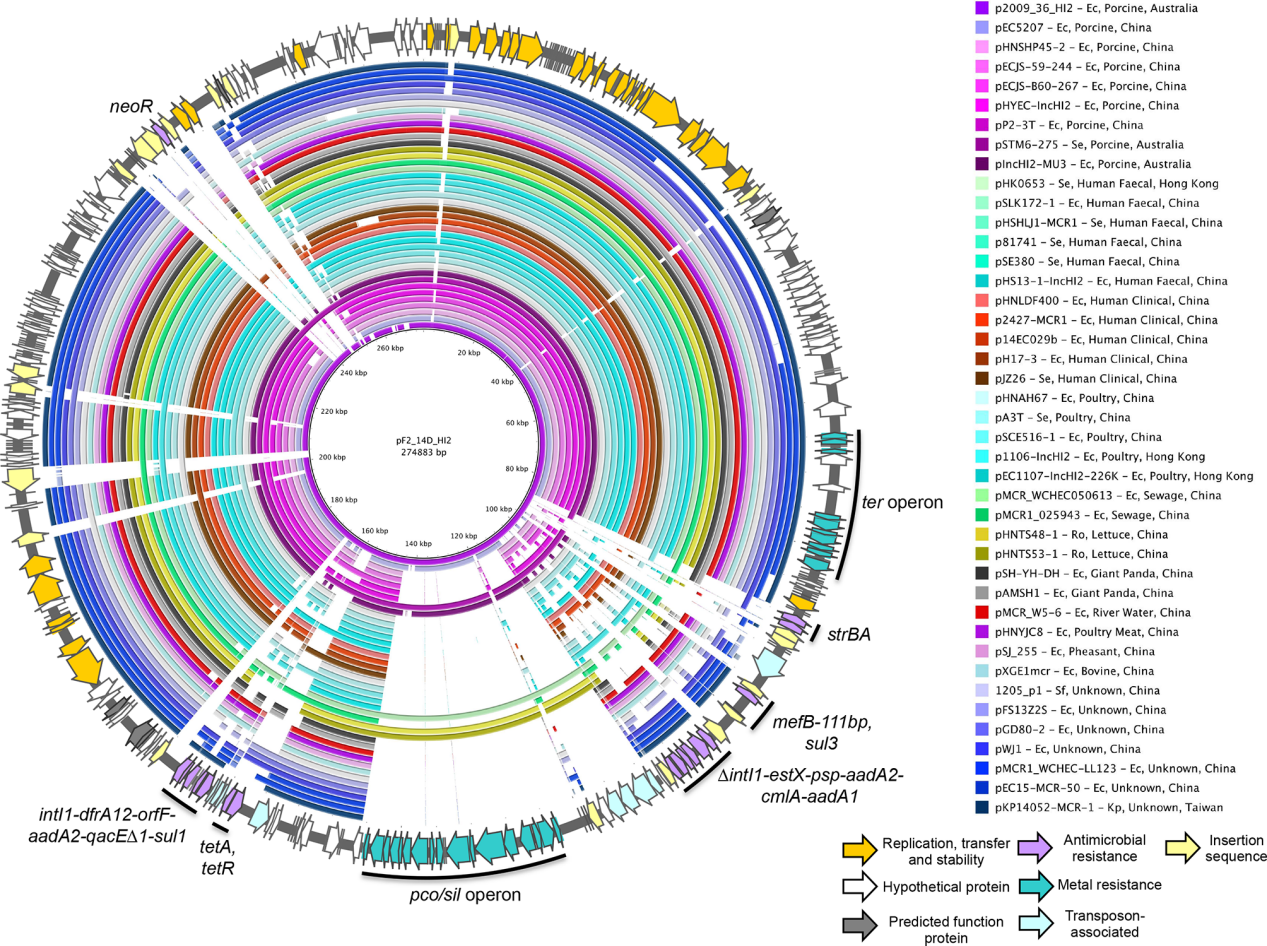
Schematic map of pF2_14D_HI2 (outer ring) and BRIG compared to p2009_36_HI2 and 41 HI2-ST3 complete plasmids. Coloured arrows represent genes and their functions. Coloured rings indicate regions of sequence homology to pF2_14D_HI2 and white space indicates an absence of homologous sequence. Host species abbreviations are as follows: Ec, *Escherichia coli *; Se, *Salmonella enterica *; Ro, *Raoultella ornitholytica*; Sf, *Shigella flexneri *; Kp, *Klebsiella pneumoniae.*

p2009_36_HI2 and pF2_14D_HI2 are large HI2-ST3 antimicrobial and metal resistance plasmids; p2009_36_HI2 and pF2_14D_HI2 were 278 665 and 274 883 bp long respectively and shared 99 % sequence identity across 98 % of their length. Most of the differences in gene content were due to acquisition of insertion sequences (IS) and truncations associated with IS activity. Relative to pF2_14D_HI2, p2009_36_HI2 carried five additional copies of IS*1203*-like elements, six additional copies of IS*26* and a copy of IS*1294.* These IS elements appear responsible for rearrangements in the backbone of p2009_36_HI2. Due to multiple insertions, rearrangements and a lack of matching IS-associated direct repeats it is difficult to accurately determine the process of evolution that led to the structure of p2009_36_HI2, although it appears that one or more insertions of IS*26* has led to a large inversion relative to pF2_14D_HI2 (Fig. 2) [59]. Aside from these differences, both plasmids were remarkably similar. Both carried two identical class 1 integron structures (Figs 2 and 3). The first of these was RR1, an IS*26* flanked *sul3-*associated integron lacking direct repeats, suggesting that an IS*26*-related homologous recombination event mediated its insertion. It consisted of ∆*intI1* truncated by a copy of IS*26*, followed by a cassette array *estX-psp-aadA2-cmlA-aadA1.* The 3′ conserved sequence (3′-*CS*) consists of IS*1203* inserted within *qacH,* followed by putative transposase *tnp440* and a downstream module IS*26-∆mefB-orfB-orfA-sul3-*IS*26.* Only 111 bp of the macrolide efflux gene *mefB* remained. This deletion signature was common in the collection from which F2_14D was sourced and all other strains carrying it also carried an HI2 replicon [20]. This suggests strongly that plasmids related to pF2_14D_HI2 are responsible for the carriage of this integron in that collection. Related *sul3* integrons have also been reported in F and I1 plasmids, although this *mefB* signature has only been reported in Australian pigs to date [20, 47, 60, 61].

The second resistance region RR2 is a *sul1* integron located downstream of a Tn*1721-*like structure carrying *tetA* (Figs 2 and 3). It carries a cassette array *dfrA12-orfF-aadA2* followed by 3′-*CS* in which *tniA* is truncated by IS*26.* This integron and its relatives have been widely reported [62]. The *pco/sil* operon, conferring copper and silver resistance, associated with a suite of Tn*7*-like transposase genes was present in both plasmids as well as the *ter* gene cluster conferring tellurite resistance [63]. p2009_36_HI2 and pF2_14D_HI2 are closely related to HI2-ST3 resistance plasmids circulating in the Asia-Pacific region

Using the pMLST alleles as a query, we downloaded all 41 complete HI2-ST3 plasmid sequences for comparison with pF2_14D_HI2 and p2009_36_HI2 (Fig. 3 and Table S2). The sequences originated from China (*n*=35), Hong Kong (*n*=3), Australia (*n*=2) and Taiwan (*n*=1), reflecting numerous reports indicating they are endemic to the Asia-Pacific region [63]. The two non-Chinese sequences were both from Australian pigs, one originating from *E. coli* and the other from a *Salmonella enterica* subspecies. The presence of HI2-ST3 in human faeces, infections, swine, poultry, sewage, lettuce and even giant panda suggests these plasmids provide a fitness advantage to gram-negative bacteria across a wide variety of niches. Furthermore, carriage in multiple *E. coli* lineages*, Klebsiella pneumoniae, Raoultella ornitholytica* and *S. enterica* subsp*.* indicates they are frequently transferred within and between species relevant to human health [60, 64–73]. Together, these data show there is a need for understanding local patterns in antimicrobial resistance plasmid carriage to better manage the risks associated with their acquisition by pathogens such as ST131.

The HI2-ST3 backbone included conjugative transfer genes, a tellurite resistance operon and numerous hypothetical proteins. The backbone appeared to be highly conserved across all sequences. Differences between plasmids were due primarily to ISs and drug resistance regions (Fig. 3). The presence of the *pco/sil* resistance operon in tandem with ARGs in all three Australian porcine strains is concerning. The use of copper as an in-feed additive for production swine and use of antimicrobials for disease control provides a dual selection pressure for these plasmids and explains their carriage in porcine populations of *E. coli* [74, 75]. This combination of heavy metal and antimicrobial resistance lends HI2 plasmids to success in wastewater environments, which are heavily contaminated with such compounds. Despite the fact there was only one HI2 plasmid from wastewater among the publicly available plasmids, wastewater should be investigated for the presence of HI2-ST3 plasmids. Wastewater in general requires further study with respect to antimicrobial resistance, as it may be an important driver of MDR plasmid evolution [76, 77]. Furthermore, HI2 plasmids are known to conjugate more effectively at 27 °C than at 37 °C, suggesting that despite their carriage in animals, the majority of conjugative transfer events may occur in environmental niches such as wastewater [60]. Further work is required to determine the full range and transmission of HI2-ST3 in the Asia-Pacific region, and these plasmids should be monitored in the context of human health.

**Fig. 4.**
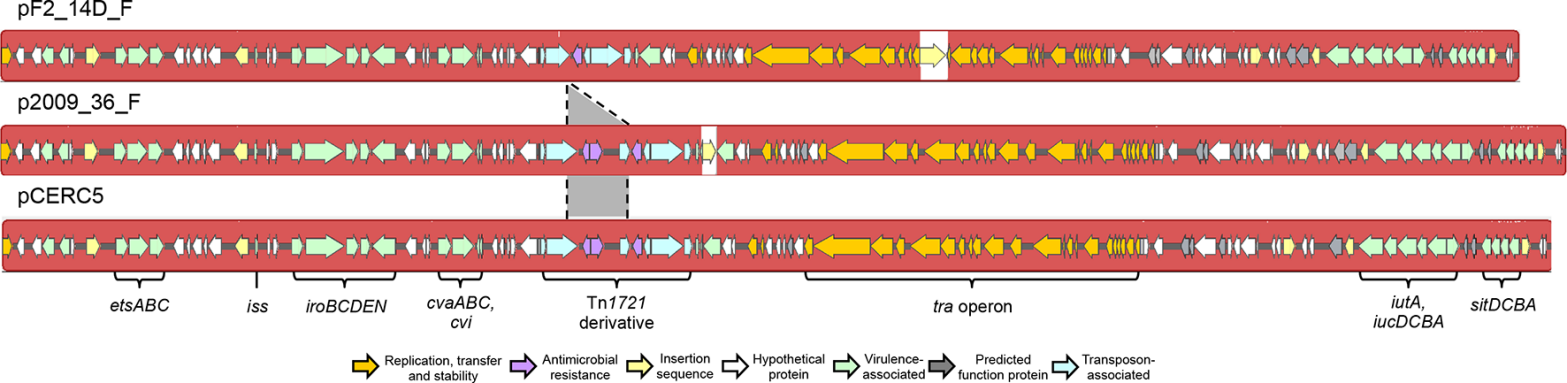
Mauve comparison of pF2_14D_F, p2009_36_F and pCERC5. Coloured blocks indicate regions of sequence homology, and white space indicates absence of homology. Grey colouring between plasmids shows deletion in F2_14D_F. Coloured arrows represent genes and their functions.

**Fig. 5.**
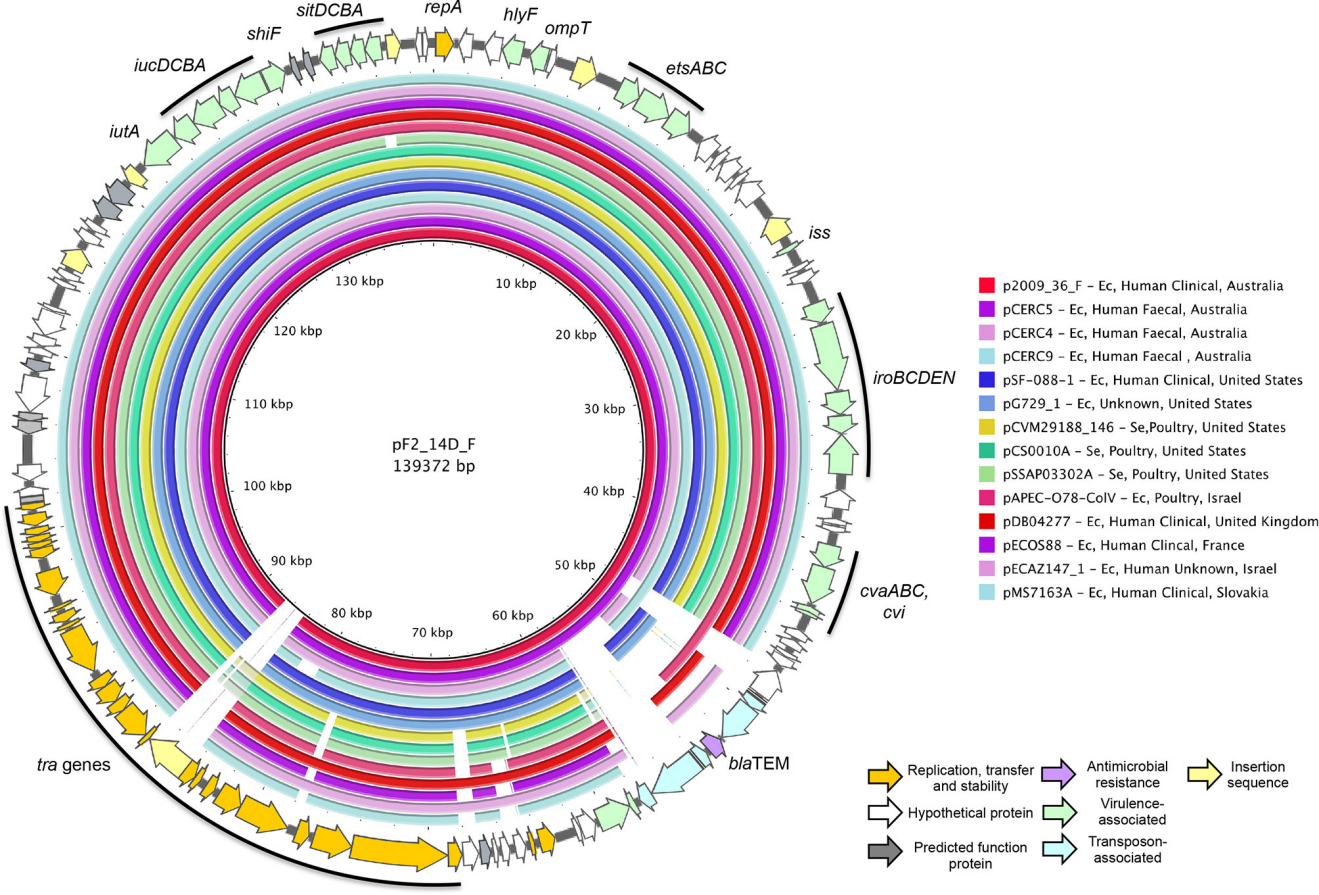
Schematic map of pF2_14D_F (outer ring) and BRIG compared to p2009_36_F and 13 F2:B1 complete plasmids. Coloured arrows represent genes and their functions. Coloured rings indicate regions of sequence homology to pF2_14D_F and white space indicates absence of homologous sequence. Host species abbreviations are as follows: Ec, *Escherichia coli*; Se, *Salmonella enterica.*

2009_36 and F2_14D carry closely related ColV F2:B1 virulence plasmids associated with pathogenicity, and p2009_36_F and pF2_14D_F are ColV virulence plasmids with identical F2:B1 replicons, 143 671 and 139 372 bp in length respectively. pF2_14D_F carried a copy of IS*Ec23* that was absent from p2009_36_F whilst p2009_36_F carried an IS*1203-*like element that was absent from pF2_14D_F. Both plasmids carried Tn*2,* inserted within a Tn*1721-*derivative transposon, although the *tet* genes and part of *tnpA-1721* were deleted in pF2_14D_F. Otherwise, these two plasmids were identical in structure (Figs 4 and 5). They carried *cvaABC* and *cvi* colicin genes, as well as numerous VAGs including the *iroBCDEN* salmochelin operon, *etsABC* type 1 secretion system, *iucABCD* and *iutA* aerobactin operon, *hlyF, ompT* and *iss* [46, 78, 79].

We queried GenBank with the shared backbone of the two plasmids, free of IS elements and Tn*1721* derivatives, and downloaded complete plasmids with ≥97 % sequence homology across their length for comparison (Fig. 5, Table S3). Sequences originated from human faecal, clinical and poultry sources, reflecting previously reported reservoirs where ColV plasmids may confer a selective advantage. The geographical distribution included Australia, Europe and North America, but Asia was not represented. Our plasmids were most similar to the pCERC plasmids from human faecal *E. coli* described by Moran and Hall [36, 47]. pCERC5 was the most similar, carrying the same backbone and Tn*1721-*derivative as p2009_36_F, the only difference being an additional insertion of an IS*1203*-like element in p2009_36_F (Figs 4 and 5). Remarkably, pCERC5 was isolated from 11.3-R3, the closest relative of our two strains on the SNP tree.

### Transfer of strains and plasmids

The nearly identical core genomes and plasmid carriage of F2_14D, 2009_36 and 11.3-R3 are intriguing, and raise more questions than they answer. First, the conserved carriage of F2:B1 ColV plasmids in a limited evolutionary background indicates that a common ancestor of these strains acquired this plasmid. For a number of reasons, we believe that the HI2-ST3 plasmid has evolved within or in close proximity to pig production and subsequently been heavily selected before disseminating beyond the production facility. First, most HI2-ST3 plasmids do not carry the *pco/sil* operon. p2009_36_HI2 was the only human clinical sequence examined that possessed this locus. The others were from Australian pigs and Chinese lettuce and sewage. Heavy metal resistance is likely to be enriched in all three of these settings by the selective pressure of copper in pig feed and animal manure that is used to fertilize crops, as well as heavy metal contamination of sewage [75, 77, 80]. Second, the *sul3* integron with identical *mefB* deletion is a genetic signature that, to date, has only been reported in Australian pigs, suggesting it evolved and disseminated from this niche [20, 60]. Finally, the extensive IS-mediated rearrangement in p2009_36_HI2 indicates it has evolved from pF2_14D_HI2 or a very similar plasmid, possibly in a hospital environment [81]. Whether the presence of such a closely related strain and plasmid in a human infection is attributable to a direct ‘food chain–human gut–urinary tract’ pathway or one involving multiple intermediary reservoirs cannot be determined with current data. It is possible that the ancestor of both strains was introduced to pigs via humans, although the presence of the HI2-ST3 plasmid and its specific gene carriage suggests subsequent backflow from pigs to humans. We concede that far more long-read sequencing of closely related strains is required to confirm this hypothesis; however, the observations and implications emerging from such a small dataset are quite surprising. Whilst animal production may not be the true origin of human-adapted pathogens such as ST131, it may nonetheless play an ongoing role in their evolution.

## Data bibliography

See references [31-34].

## Supplementary Data

Supplementary File 1

Supplementary File 2

Supplementary File 3
