# Supplementary material for: Clonal ST131-H22 Escherichia coli strains from a healthy pig and a human urinary tract infection carry highly similar resistance and virulence plasmids
Source: Microb Genom. 2019 Sep 17;5(9):e000295. doi: 10.1099/mgen.0.000295 (PMC6807379; doi:10.1099/mgen.0.000295)
Supplement: Supplementary File 1 [file mgen-5-295-s001.pdf]

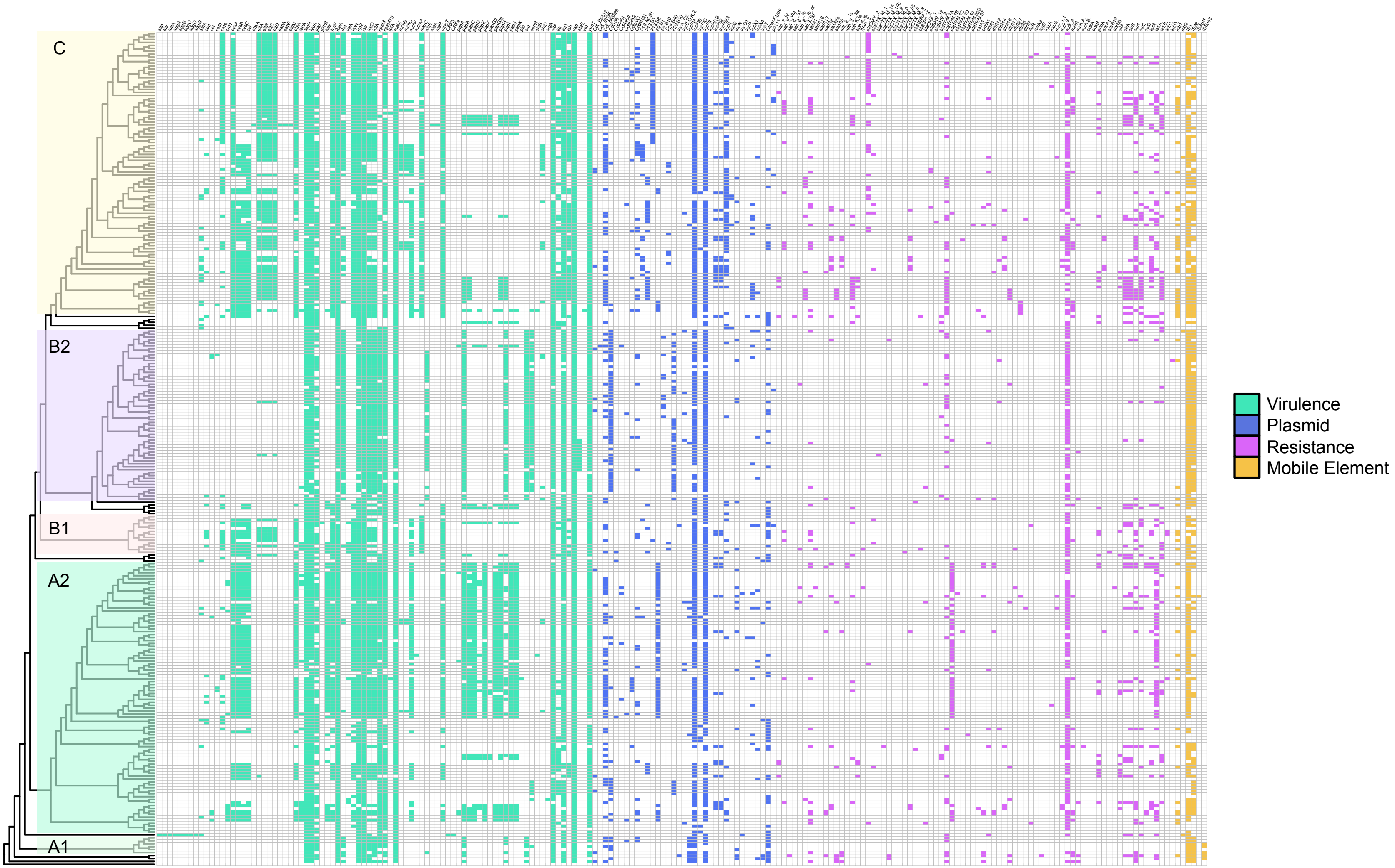

Fig. S1. Maximum-likelihood phylogenetic as seen in Fig. 1, mapped against gene presence/absence for virulence, plasmid, antimicrobial resistance and mobile element genes. Clades are labelled as in Fig. 1
