# Supplementary material for: Clonal ST131-H22 Escherichia coli strains from a healthy pig and a human urinary tract infection carry highly similar resistance and virulence plasmids
Source: Microb Genom. 2019 Sep 17;5(9):e000295. doi: 10.1099/mgen.0.000295 (PMC6807379; doi:10.1099/mgen.0.000295)
Supplement: Supplementary File 3 [file mgen-5-295-s003.pdf]

Extracting DNA using SDS cell lysis followed by phenol-chloroform

Before starting:

1. Set heat blocks to 37 and 56 degrees C.
2. Check you have the following reagents/solutions
    a. TE
    b. 20% SDS
    c. Proteinase K
    d. RNase A
    e. Ethanol 70% and 100%
    f. Phenol/chloroform/isoamyl alcohol
    g. Chloroform/isoamyl alcohol
    h. 3M sodium acetate pH5.2

1. Prepare an overnight culture in rich media.

2. Sub-culture 100uL of this overnight culture in 10mL fresh media and incubate for 3-3.5 hours.

3. Spin mid-log cells for 15 minutes at 4000 rpm and re-suspend in 500uL of Lysis buffer:

482uL	TE (pH8.0)
15uL	20% SDS
3uL	Proteinase K (20mg/mL)

4. Incubate this tube at 56°C with mixing via tube inversion every 15 minutes until completely lysed.

5. Add 1/10 volume (50ul) 3M sodium acetate pH5.2 and 2.5x volume (1.25mL) 100% ethanol and mix gently.

6. With a pipette tip, transfer the precipitated proteins and nucleic acids into a tube containing 500uL 70% ethanol.

7. Spin tubes at 13000 rpm for 10 minutes, pour off supernatant. Resuspend pellet in 800uL water (ideally pre-heated to 65°C).

8. Add 10uL of RNase A, incubate at 37°C for 30 minutes.

9. Bring the tube back to room temperature after incubation and move on with Phenol-Chloroform extraction.

10. Pre-mix phenol/chloroform/isoamyl alcohol solution (400uL of phenol; 400uL chloroform/isoamyl alcohol) and add (total 800uL).

11. Invert 6-8 times then spin at 13000 rpm for 5 minutes.

12. Remove as much liquid as possible from the top aqueous phase and place into a new tube. Avoid picking up any of the phenol/chloroform/isoamyl alcohol phase.

13. Add equal volumes of the chloroform/isoamyl alcohol solution to extracted aqueous phase.

14. Invert 6-8 times then spin at 13000 rpm for 5 minutes.

17. Remove as much of the top aqueous phase as possible and place into a new tube. Avoid picking up any of the chloroform/isoamyl alcohol phase.

18. Repeat steps 5 and 6 (ethanol precipitation, **adjust volume of sodium acetate and ethanol based on how much aqueous phase you get**).

19. Spin for 20 minutes at 13000 rpm (optionally at 4°C).

20. Decant supernatant carefully without disturbing the pellet.

21. Wash by adding 500uL of 70% ethanol and invert 6-8 times.

22. Spin for 15 minutes at 13000 rpm (optionally at 4°C).

23. Decant supernatant carefully without disturbing the pellet.

24. Repeat the wash steps (steps 21-23)

25. Quick spin on table top centrifuge to draw residual ethanol to bottom.

26. Remove residual ethanol with a p20 pipette, be careful not to disturb the pellet.

27. Air dry pellet and resuspend with appropriate volume (100uL) of pre-heated 65°C water.
